# Analysis of intraflagellar transport in the zebrafish model

**DOI:** 10.1186/2046-2530-1-S1-O11

**Published:** 2012-11-16

**Authors:** C Zhao, Y Omori, K Brodowska, P Kovach, J Malicki

**Affiliations:** 1 Tufts University, USA; 2 Osaka Biosciences Institute, Osaka, Japan; 3 Department of Biomedical Science and the MRC Centre for Developmental and Biomedical Genetics, University of Sheffield, Sheffield, UK

## 

The formation and function of cilia requires that proteins are translocated by intraflageller transport (IFT) along microtubules that support ciliary structure. Using tools of genetic analysis in zebrafish, we studied both motors that drive IFT and proteins that mediate the interaction of the IFT particle with presumptive cargo molecules. Our studies demonstrate that IFT particle components, and a Meckel-Gruber Syndrome 1 (MKS1)-related, B9 domain protein, B9d2, bind each other and contribute to the ciliary localization of Inversin (Nephrocystin 2). B9d2, Inversin, and Nephrocystin 5 support, in turn, the transport of a cargo protein, Opsin, but not another photoreceptor ciliary transmembrane protein, Peripherin. In parallel, we studied motors that drive the movement of the IFT particle, and found that kinesin 2 family motors, *kif3* and *kif17*, display very different contributions to ciliogenesis. While *kif17* appears largely dispensable, the *kif3b* gene is necessary for cilia differentiation in most tissues, although exceptions exist, and include photoreceptors and a subset of hair cells. Cilia of these cell types persist even in *kif3b*/*kif17* double mutants. In contrast to *kif3b*/*kif17* double homozygotes, simultaneous interference with *kif3b* and *kif3c* leads to the complete loss of photoreceptor and hair cell cilia, revealing redundancy of function. Moreover, our data suggest that the repertoire of kinesin motors changes in photoreceptors during their differentiation. These studies reveal molecular mechanisms that mediate the transport of ciliary proteins, and are of fundamental importance for the formation and function of several vertebrate organs.

